# An Open Time Perspective and Social Support to Sustain in Healthcare Work: Results of a Two-Wave Complete Panel Study

**DOI:** 10.3389/fpsyg.2020.01308

**Published:** 2020-06-17

**Authors:** Annet H. de Lange, Karen Pak, Eghe Osagie, Karen van Dam, Marit Christensen, Trude Furunes, Lise Tevik Løvseth, Sarah Detaille

**Affiliations:** ^1^Department Work and Organizational Psychology, Faculty of Psychology, Open University, Heerlen, Netherlands; ^2^Human Resource Management, HAN University of Applied Sciences, Nijmegen, Netherlands; ^3^Department of Psychology, Norwegian University of Science and Technology, Trondheim, Norway; ^4^Norwegian School of Hotel Management, University of Stavanger, Stavanger, Norway; ^5^Institute of Management Research, Radboud University Nijmegen, Nijmegen, Netherlands; ^6^Department of Psychiatry, St. Olav’s University Hospital, Trondheim, Norway

**Keywords:** future time perspective, longitudinal research, psychosocial work characteristics successful aging at work, sustainable employability, work ability

## Abstract

Based on lifespan developmental psychology and psychosocial work characteristics theory, we examined longitudinal relations between calendar age, occupational time perspective, different types of job demands and job resources in relation to sustainable employability (i.e., work ability, vitality and employability) among healthcare workers in Netherlands (*N* = 1478). Results of our two-wave complete panel study revealed satisfactory fit indices for the metric invariance of the included variables across the two waves (6-month time lag). Our results revealed a negative relation between calendar age and external employability of healthcare workers (limited support for hypothesis 1), and more consistent evidence for positive relations between an open future time perspective and across-time changes in vitality, work ability and external employability (supporting hypothesis 2). Few significant findings were found for relations between specific job demands or job resources and indicators of sustainable employability of healthcare workers (mixed results hypotheses 3 and 4). Our explorative tests of possible moderating effects of age or occupational time perspective in predicting relations between psychosocial work characteristics and indicators of sustainable employability revealed only a significant interaction effect of supervisor support and future time perspective in explaining across-time changes in external employability of healthcare workers (rejecting hypothesis 5 and confirming hypothesis 6). We discuss the practical as well as theoretical implications of these findings, and present recommendations for future research.

## Introduction

Within the healthcare sector in the Western society, various labor market trends have the potential to impact the quality of healthcare provided by available staff. One of the most important trends and developments among them are the graying, as well as dejuvenation of the available workforce, resulting in an overall decreasing number of healthcare staff (Herkes et al., 2019; Prins et al., 2019). Furthermore, a growing number of patients with health problems make use of the healthcare sector, implying increased numbers of patients and work pressure for the aging group of healthcare staff in coming years (Van Dam et al., 2017; Teoh et al., 2019). Consequently, work in healthcare is characterized by high levels of physical, emotional and mental demands, making the work quite taxing and stressful (Tomietto et al., 2019).

As a result, an increasing percentage of healthcare workers report serious mental health problems (Nonnis et al., 2017; Herkes et al., 2019) and find it more difficult to continue working in their profession until the official retirement age. Some of these healthcare workers decide to leave their job and transfer to another sector to find less demanding work (Boumans et al., 2008). While the need for healthcare workers increases, the number of workers is therefore likely to decline owing to the increased workload and decreased work ability of healthcare workers. This situation is urgent for healthcare organizations and society at large and it is imperative to identify factors that can facilitate the sustainable employability of healthcare workers to prolong their careers within healthcare (e.g., De Lange et al., 2015; Osagie et al., 2019).

Fortunately, a growing number of studies focuses on the antecedents of successful aging at work and sustainable employability to prolong working lives of workers in different sectors (e.g., De Lange et al., 2015; Kooij, 2015). Several definitions of successful aging have been presented in earlier research. For example, Zacher et al. (2018a) state that employees age successfully at work if they sustain the same level or deviate in increasingly positive ways from average developmental trajectories in subjective and objective work outcomes (i.e., work ability) across the working lifespan and maintain a person-job fit across time (Kooij, 2015; Zacher, 2015). More recently, De Lange et al. (2020) stated that successful aging refers to the fact that employees can pro-actively recover and improve over time through self-management, skills and actions, and with support or interventions from the work environment.

Considering new research on successful aging at work, De Lange et al. (2020) emphasize that more research can include the influence of individual difference variables that are related to aging, like an open future time perspective, to better explain developments in worker outcomes across time. This is in line with suggestions of Zacher et al. (2018a), who noted that the current body of research on successful aging at work has not yet examined the influence of individual difference variables like the experienced future time perspective in predicting sustainable employability of workers across time (Weigl et al., 2013; Baltes et al., 2014; Pak et al., 2018).

Furthermore, only a few studies on sustainable employability have been conducted in healthcare settings and no longitudinal study to date has been conducted on the influence of future time perspective in relation to sustainable employability of healthcare workers (Rudolph et al., 2018; Zacher et al., 2018a). Such insights are crucial to better understand the underlying mechanisms in successful aging and sustainable employability of healthcare workers. Increased insights in the influence of individual difference variables like time perspective will enable employers to stimulate successful aging more effectively and better intervene if necessary.

The current study is the first multi-wave study that aims to overcome the aforementioned research gaps by formulating and testing new theory-based hypotheses for relations between aging, time perspective and indicators of sustainable employability in healthcare work. As a result, the results of this new two-wave complete panel study can provide new insights into the question on how to better sustain aging workers in healthcare. Before we present the hypotheses of our study, we will first pay attention to the concepts and related theories addressing the topic of sustainable employability, the factor of time perspective and the influence of psychosocial work. We will start with describing the indicators of sustainable employability.

### Sustainable Employability: Vitality, Work Ability, and Employability

#### Sustainable Employability

Several important aspects of sustainable employability have been distinguished by social partners (SER, 2009) and researchers (De Lange et al., 2015; De Vos and Van der Heijden, 2015; Semeijn et al., 2015; Van der Klink et al., 2016; Van Dam et al., 2017). These aspects include: (i) work ability (Ilmarinen, 2007), (ii) vitality (Schaufeli et al., 2006; Bakker and Demerouti, 2017), and (iii) employability (Fugate et al., 2004; Van der Heijde and Van der Heijden, 2006). These aspects of sustainable employability relate to human strengths, health, and motivation in organizations and are considered essential for employees to sustain their performance at work (Semeijn et al., 2015; Van Dam et al., 2017).

More specifically, work ability represents the health component of sustainable employability, and is defined as the extent to which one is physically and mentally able to keep performing one’s job now and in the future (Ilmarinen et al., 2005; Ilmarinen, 2006, 2007; Van Vuuren, 2012). Furthermore, vitality represents the motivational component of sustainable employability and is characterized by high levels of energy and mental resilience while working, and the willingness to invest effort in one’s work, and persist even in the face of difficulties (Schaufeli et al., 2006). Within the Job Demands-Resources model (JD-R), vitality is an important component of work engagement (Bakker and Demerouti, 2017; Bakker, 2018; Bakker et al., 2019).

Employability refers to the individuals’ opportunity to retain or find work inside and outside of the current organization (Van Dam et al., 2017; Van der Heijde and Van der Heijden, 2006). Although the opportunity to retain or find work might depend on labor market characteristics, it is generally noted that individuals’ characteristics, such as their abilities, skills, and knowledge, contribute to employability and labor market participation (Berntson et al., 2006; Semeijn et al., 2015).

#### Sustainable Employability and Aging Healthcare Workers

Healthcare workers’ sustainable employability is likely to decline when they age. Although individual differences may exist, the aging process of employees is generally accompanied by decreases in physical and mental capacities (Ilmarinen, 2006; Truxillo et al., 2015; Van der Mark-Reeuwijk et al., 2019). As the work demands in healthcare will further increase in the future, healthcare workers may be faced with even more mental or physical health challenges and thus with lowered work ability. Similarly, vitality and employability may also diminish over time. Organizations are often inclined to provide older employees with fewer learning opportunities, job changes, and challenging task assignments compared to younger employees (Furunes and Mykletun, 2010; Truxillo et al., 2015). This might relate to some persistent stereotypes as well as the risk of self-stereotyping concerning older workers’ learning motivation and capabilities, and their openness toward change (Kanfer and Ackerman, 2004; Ng and Feldman, 2012; Finkelstein et al., 2013). Therefore, older workers can find themselves stuck in repetitive jobs with little learning potential, which may undermine their employability and motivation (Truxillo et al., 2012; Van Dam et al., 2017). Moreover, older employees often perceive fewer labor market opportunities, which can lower their employability perceptions (Rothwell and Arnold, 2007).

Previous studies have indeed found negative relationships between calendar age and the three aspects of sustainable employability (see for example Ilmarinen, 2007; Monteiro et al., 2006; Van den Berg et al., 2009; Van Dam et al., 2017). For instance, Ilmarinen (2007) observed differences in the development of work ability in different age groups and different types of occupations. Studying workers in a public health institution, Monteiro et al. (2006) found that higher age, lower education (i.e., employability), and long work history in the organization were associated with reduced work ability. Van Dam et al. (2017) observed that older employees generally reported lower employability, while only those who had challenging and rewarding jobs reported similar employability levels as their younger colleagues. Similarly, Van Vuuren et al. (2011) found that work ability and employability generally declined with age, while this effect did not occur for those employees who were provided with ample opportunities for formal and informal learning (see also Van der Heijden et al., 2015). Based on these studies, we expect to find negative relations between calendar age and indicators of sustainable employability (i.e., vitality, work ability, and employability; *Hypothesis 1*). Another important individual difference variable is future time perspective.

### Socioemotional Selectivity Theory and Future Time Perspective: Concepts and Theory

Socioemotional Selectivity Theory (SST theory; Carstensen, 2019) describes the motivational consequences of a changing “temporal horizon” as people age. According to SST theory individuals will select goals in accordance with their perceptions of the future as being limited or open-ended (Lang and Carstensen, 2002). More specifically younger people perceive time as open-ended (holding a “time since birth” perspective) and will therefore be especially motivated by growth or knowledge-related goals (new information or social interactions) that may be useful in their future. In contrast, older people perceive time as a constraint (holding a “time till death” perspective) and will be more motivated by achieving short-term emotion-related goals, such as deepening one’s existing social relations. As such, future time perspective appears an important precursor of workers’ goal striving and self-management at work and is therefore an important individual factor to consider in terms of successful aging and sustainable employability. Studies have found consistent negative associations between an open occupational future perspective and calendar age as well as positive associations with work outcomes like continuance work motivation (Lang and Carstensen, 2002; Schmitt et al., 2013; Rudolph et al., 2018). In short, the socioemotional selectivity theory has received empirical support in many experimental as well as field studies (Henry et al., 2017; Rudolph et al., 2018). The results of this body of research indicates that especially an open occupational time perspective is associated with indicators of sustainable employability. In this study we will test whether this hypothesis is true for healthcare workers. Moreover, we will test whether this association holds over time. That is, whether future time perspective can predict across-time changes in sustainable employability of healthcare workers (*Hypothesis 2*).

### Psychosocial Work Characteristics

Zacher et al. (2018b) stress the importance of paying attention to the pivotal role of psychosocial work characteristics in explaining developments in sustainable employability of aging workers across time (see also Truxillo et al., 2015). According to the Job Demands-Resources (JD-R) model (Bakker and Demerouti, 2017; Bakker, 2018) psychosocial work characteristics can best be measured by a combination of job demands and job resources. Job demands refer to “physical, psychological, social, or organizational aspects of the job that require sustained physical and/or psychological (cognitive and emotional) effort or skills and are therefore associated with certain physiological and/or psychological costs” (Bakker and Demerouti, 2017, p. 312). Examples of job demands are physical or mental demands or workload. Job resources are defined as the “physical, psychological, social, or organizational aspects of the job that are either/or functional in achieving work goals, reduce job demands and the associated physiological and psychological costs and stimulate personal growth, learning, and development” (Bakker and Demerouti, 2017, p. 312). Examples of job resources are pay, supervisor support, and autonomy. The JD-R model (Bakker and Demerouti, 2017; Vagharseyyedin, 2016; Bakker, 2018) suggests that job demands have a negative effect on employee outcomes as they trigger a health impairment process; having too much job demands deplete one’s personal resources and lead to exhaustion. Job resources, on the other hand, have a positive effect on work outcomes as they trigger a motivational process (Bakker, 2018). More recently, Pak et al. (2018) also confirmed these findings in their systematic review of 110 empirical studies examining relations between job characteristics and indicators of sustainable employability. As a consequence, we expect to replicate these results in this new longitudinal study among healthcare workers, and hypothesize that job demands will have a significant negative relation with indicators of sustainable employability (*Hypothesis 3*), and job resources will have a positive relation with indicators of sustainable employability (*Hypothesis 4*).

#### Interaction Effects Age-Related Variables, Job Demands, Job Resources

Previous studies suggest that individual factors and contextual factors interact in determining work behavior and outcomes (Johns, 2006; Withagen et al., 2012). Therefore, it is possible that calendar age and occupational future time perspective, in addition to their direct impact, will moderate the relationships between the psychosocial work characteristics and indicators of sustainable employability (Zacher and Schmitt, 2016). Healthcare workers’ age might be especially relevant for the impact of job demands. As workers age, their psychological and physical resources may decline while their ability to recover is reduced (Kiss et al., 2008; Truxillo et al., 2015). As such, the impact of high job demands will be larger for older workers than for younger workers. This will be especially true for healthcare professionals who are faced with high emotional and physical demands. Research provides general support for an interaction effect of age with work characteristics on workers’ well-being, vitality, and employability (Zacher and Schmitt, 2016; Van Dam et al., 2017). Therefore, it is expected that the negative relationships of job demands with sustainable employability are stronger for older workers than for younger workers (*Hypothesis 5*).

Similarly, future occupational time perspective might moderate the relationships of job resources with sustainable employability, such that job resources will contribute more to sustainable employability for those healthcare workers with an open future time perspective (*Hypothesis 6*). This is in line with SST’s claim that future time perspective is an important precursor of workers’ goal striving and self-management at work. Only a few studies have focused on a possible moderating role of future time perspective. For instance, Schmitt et al. (2013) found that future time perspective moderated the relationship of autonomy with work engagement.

Summarizing, we will test the following hypotheses in this two-wave complete panel study among healthcare workers:

• Hypothesis 1: Calendar age is negatively related to indicators of sustainable employability (i.e., vitality, employability, and work ability).
• Hypothesis 2: Future time perspective is positively related to indicators of sustainable employability (i.e., vitality, employability, and work ability).
• Hypothesis 3: Job demands (i.e., workload, physical demands, emotional demands, and mental demands) are negatively related to indicators of sustainable employability (i.e., vitality, employability, and work ability).
• Hypothesis 4: Job resources (i.e., autonomy, supervisor support, and colleague support) are positively related to indicators of sustainable employability (i.e., vitality, employability, and work ability).
• Hypothesis 5: Calendar age moderates the relations between job demands (i.e., workload, physical demands, emotional demands, and mental demands) and indicators of sustainable employability of healthcare workers (i.e., vitality, employability, and work ability).
• Hypothesis 6: Future time perspective moderates the relations between job resources (i.e., autonomy, supervisor support, and colleague support) and indicators of sustainable employability of healthcare workers (i.e., vitality, employability, and work ability).

## Materials and Methods

### Design of the Study and Procedure

This study is embedded in a larger research project among 25 healthcare institutions in Netherlands, referred to as “the Healthy Healthcare project” that emphasizes a system-based understanding of the interrelation between organizational structure, workers health and quality of patient care. Within this project, data was collected longitudinally using a questionnaire at T1 and T2 with a mean time lag of 6 months (i.e., a panel design). This length of time lag is in line with the recommendations of Dormann and Griffin (2015) of a relatively short time lags in survey research focusing on psychosocial work characteristics and worker outcomes. Moreover, a time lag of 6 months was considered appropriate as previous studies have demonstrated that the outcomes included in this study can fluctuate in rather short periods (De Lange et al., 2004; Zacher et al., 2014; Akkermans et al., 2019; Rudolph and McGonagle, 2019; Rudolph and Zacher, 2020). The first questionnaire (T1) was sent to the participants between November 2017 and the end of January 2018, the second questionnaire (T2) between June 2018 and the middle of August 2018.

### Sample

The 25 healthcare institutions included in this study mainly focused on elderly care, care for the disabled and home care, but also included facilities for addiction treatment, youth services, mental health care and home care. The final sample consists of the 1478 employees from these institutions who completed the questionnaire at both the first (T1; 2967 of 6866 employees, response rate = 39.3%) and the second measurement moment (T2; 2132 employees, follow-up response rate = 71,9%). *M*_age_ = 46.8 years (*SD* = 11.06 years), range = 18–58 years, with most respondents being female (84%, *n* = 1242), with fixed contracts (89.6%, *n* = 1325). Vocational education (37.8%, *n* = 558) and a bachelor’s degree (35.7%; *n* = 527) were the most common education levels. 779 employees held a healthcare position (52.7%) with the remainder working in a leadership or support functions. Caregiver was the most common job category (15.1%), followed by leader (6.9%), nurse with a vocational degree (6.4%), and pedagogical employee (5.1%).

### Measures

#### Work Ability

The Work Ability Index (WAI; Ilmarinen, 2006) was used to measure *work ability*. The WAI consists of seven constructs (60 items in total): i.e., (1) current work ability, (2) work ability in relation to the physical and mental demands of the job, (3) current diseases, illnesses, and injuries, (4) limitations due to diseases, illnesses, and injuries, (5) sick leave, (6) future expectation of work ability, and (7) mental resources. Although work ability is measured as a multidimensional construct with the WAI, it is mainly employed as a unidimensional construct in most studies and in practice, and the healthcare practice in Netherlands in particular (see Osagie et al., 2019 for a review). So, for comparison and recognition reasons, we will also address it as a unidimensional construct in the current study. An example item from the WAI is “*Assume that your work ability at its best has had a value of 10. How many points would you give your current work ability*?” Scores on each dimension were summed, with a minimum score of 7 and a maximum score of 49. In our sample, work ability scores ranged from 14.5 to 49 at T1 and from 12 to 49 at T2 with a median of 42 at both time points.

#### Vitality

*Vitality* was measured with three items of the shortened Utrecht Engagement Scale (Schaufeli et al., 2006). Items were measured on a six-point Likert scale ranging from ‘never’ (1) to ‘daily’ (6). An example item is “*At my work, I feel bursting with energy.*”

#### Internal and External Employability

Internal and external employability were measured with the eight items scale of De Cuyper and De Witte (2008) using a five-point Likert scale ranging from ‘completely disagree’ (1) to ‘completely agree’ (5). Four items covered *internal employability* (An example item: “I am able to get different jobs with my current employer”) and four items covered *external employability* (An example item: “I would be able to find a different, equivalent job”).

#### Job Demands and Resources

Job demands were measured with four scales from the VBBA (Van Veldhoven and Meijman, 1994) at the first and second measurement moment. *Physical demands* were measured with three items (an example item: “Does your work require physical strength?”) *mental demands* (an example item: “Do you have to work very precisely?”) with four items, *emotional demands* with five items (an example item: “Is your work emotionally demanding?”), and *workload* with six items (an example item: “Do you need to rush at work?”). All items were measured on a four-point scale ranging from ‘always’ (1) to ‘never’ (4) and recoded in the opposite direction to facilitate interpretation.

#### Job Resources

Job resources were measured with three scales of the VBBA (Van Veldhoven and Meijman, 1994) at the first and second measurement moment. *Autonomy* was measured with four items (an example item: “Can you organize your work yourself?”). *Colleague support* (an example item: “If necessary, can you ask your colleagues for help?”) and *supervisor support* (an example item: “If necessary, can you ask your direct guidance for help?”) were each measured with six items. All items were measured on a four-point scale ranging from ‘always’ (1) to ‘never’ (4) and recoded in the opposite direction to facilitate interpretation.

#### Future Time Perspective

*Occupational future time perspective* was measured with a six item scale developed by Zacher and Frese (2009) which is an adaptation of the future time perspective scale of Carstensen and Lang (1996). An example item is ‘Many opportunities await me in my occupational future.’ Items were measured on a five-point Likert scale ranging from ‘does not apply at all’ (1) to ‘applies completely’ (5).

#### Calendar Age

Age was measured at the first measurement moment as a continuous variable.

### Analyses

We conducted hierarchical regression analyses to test our hypotheses using M-Plus (version 8; see Table 3 through 6) because we aimed to predict changes in sustainable employability through the selected individual factors and work characteristics and because our hypotheses were in part explorative (e.g., the interaction hypotheses) in nature (cf. Bollen and Pearl, 2013). In the first models the control variables (i.e., the outcome variables at the first measurement moment) were included. Next, because, as mentioned before, one’s work behavior is influenced by both individual factors and contextual factors simultaneously (Johns, 2006; Withagen et al., 2012) we included both sets of variables (with workers’ experiences of job demand and job resources as a proxy for contextual factors) in the second models. In the final models (the third model to be tested) the interaction terms of job demands with age, job demands with future time perspective, job resources with age and job resources with future time perspective were added. This allowed us to examine the effects of the predictors as they occur in practice, namely interrelated and simultaneously.

## Results

To examine whether the different variables in this study captured different constructs, confirmatory factor analyses were conducted for the variables included in this study using M-Plus (version 8). In line with the recommendations of Hu and Bentler (1999) we used multiple fit indices, including the chi-square test (Δχ*^2^*), comparative fit index (CFI; Bentler, 1990), Tucker-Lewis Index (TLI; Tucker and Lewis, 1973), the root mean square error of approximation (RMSEA; Steiger and Lind, 1980) and the standardized root mean square residual index (SRMR; Hu and Bentler, 1995) to determine model fit. We compared the proposed 12-factor model at T1 (i.e., internal employability, external employability, vitality, work ability, emotional demands, mental demands, physical demands, workload, autonomy, supervisor support, colleague support, and future time perspective), with a nine factor model (i.e., Factor 1 = internal employability and external employability; Factor 2 = vitality and work ability; Factor 3 = emotional demands; Factor 4 = mental demands; Factor 5 = physical demands; Factor 6 = workload; Factor 7 = autonomy; Factor 8 = supervisor support and colleague support; Factor 9 = future time perspective) and a four factor model (i.e., Factor 1 = internal employability, external employability, vitality and work ability; Factor 2 = emotional demands, mental demands, physical demands, and workload; Factor 3 = autonomy, supervisor support, and colleague support; Factor 4 = future time perspective) and a one factor model.

We found that the 12-factor model (χ*^2^* = 3789.50, *df* = 1519, *p* < 0.001, CFI = 0.94, TLI = 0.94, RMSEA = 0.03, SRMR = 0.06) fit the data significantly better than the nine-factor model (Δχ*^2^* = 3162.16, Δ*df* = 30, *p* < 0.001), and the four-factor model (Δχ*^2^* = 14796.52, Δ*df* = 62, *p* < 0.001). The one factor model could not be converged. At the second measurement moment we compared the proposed 11 factor model (i.e., internal employability, external employability, vitality, work ability, emotional demands, mental demands, physical demands, workload, autonomy, supervisor support, and colleague support), with an eight factor model (i.e., Factor 1 = internal employability and external employability; Factor 2 = vitality and work ability; Factor 3 = emotional demands; Factor 4 = mental demands; Factor 5 = physical demands; Factor 6 = workload; Factor 7 = autonomy; Factor 8 = supervisor support and colleague support) and a three factor model (i.e., Factor 1 = internal employability, external employability, vitality and work ability; Factor 2 = emotional demands, mental demands, physical demands, and workload; Factor 3 = autonomy, supervisor support, and colleague support) and a one factor model. We found that the 11-factor model (χ*^2^* = 3281.47, *df* = 1219, *p* < 0.001, CFI = 0.91, TLI = 0.90, RMSEA = 0.03, SRMR = 0.06) fit the data significantly better than the eight-factor model (Δχ*^2^* = 3160.879.15, Δ*df* = 27, *p* < 0.001), the three-factor model (Δχ*^2^* = 11377.53, Δ*df* = 52, *p* < 0.001), and the one-factor model (Δχ*^2^* = 15662.14, Δ*df* = 55, *p* < 0.001). These results support the notion that our measures can be empirically distinguished.

Based on recommendations of Van de Schoot et al. (2012), the measurement invariance over time was examined for all outcome variables (Table 1). For vitality the requirements of configural measurement invariance were met. For employability and work ability the chi square test suggests measurement variance, however as the CFI and RMSEA test suggest invariance the requirement of configural invariance were met. The requirements for metric invariance are also met for vitality and work ability but not for employability. Next, scale scores were created for each of the variables to simplify the model. In Table 2, the correlations between the variables that are included in this study are shown as well as the Cronbach’s alphas.

**TABLE 1:** Measurement invariance.

**Variable**	**Type**	**χ^2^**	**df**	**CFI**	**RMSEA**	**Δχ^2^**	**Δdf**	***p***	**ΔCFI**	**ΔRMSEA**
Vitality	Configural	33.178	5	0.996	0.062	2.524	3	0.471	0	0.013
	Metric	35.702	8	0.996	0.049	4.507	2	0.105	0	0.003
	Scalar	40.209	10	0.996	0.046					
Employability	Configural	349.060	92	0.984	0.054	170.929	10	0.000	0.010	0.001
	Metric	519.989	102	0.974	0.055	1153.596	10	0.000	0.070	0.046
	Scalar	1673.585	112	0.904	0.101					
Work ability	Configural	520.490	71	0.927	0.063	14.956	6	0.021	0.001	0.005
	Metric	535.446	77	0.926	0.067	20.278	6	0.002	0.002	0.003
	Scalar	555.724	83	0.924	0.070					

**TABLE 2:** Means, standard deviations, correlations, and Cronbach’s alpha’s (on the diagonal line).

	***M***	***SD***	**1**	**2**	**3**	**4**	**5**	**6**	**7**	**8**	**9**	**10**	**11**	**12**	**13**	**14**	**15**	**16**	**17**	**18**	**19**	**20**	**21**	**22**	**23**
(1) Work ability T1	40.70	5.07																							
(2) Work ability T2	40.86	5.21	0.73**																						
(3) Internal employability T1	3.18	0.71	0.20**	0.17**	(0.72)																				
(4) Internal employability T2	3.20	0.71	0.19**	0.21**	0.61**	(0.73)																			
(5) External employability T1	3.12	1.01	0.25**	0.22**	0.34**	0.31**	(0.94)																		
(6) External employability T2	3.31	1.02	0.23**	0.24**	0.32**	0.36**	0.75**	(0.95)																	
(7) Vitality T1	4.46	1.09	0.43**	0.31**	0.22**	0.19**	0.16**	0.12**	(0.91)																
(8) Vitality T2	4.43	1.09	0.35**	0.42**	0.17**	0.24**	0.12**	0.12**	0.66**	(0.91)															
(9) Physical demands T1	1.86	0.86	−0.28**	−0.28**	–0.03	0.01	0.10**	0.09**	0.04	0.03	(0.93)														
(10) Physical demands T2	1.98	0.85	−0.25**	−0.32**	–0.06	0.00	0.02	0.01	–0.05	–0.06	0.84**	(0.93)													
(11) Mental demands T1	3.32	0.54	–0.03	–0.05	0.03	0.01	0.07*	0.04	0.06*	0.05*	0.05	0.03	(0.81)												
(12) Mental demands T2	3.29	0.54	0.05	0.02	–0.05	0.02	–0.04	–0.06	0.09*	0.14**	0.05	0.06	0.60**	(0.82)											
(13) Emotional demands T1	2.27	0.45	−0.18**	−0.15**	0.01	–0.03	0.13**	0.13**	−0.15**	−0.17**	0.05	0.12**	0.16**	0.11*	(0.77)										
(14) Emotional demands T2	2.23	0.41	–0.08	−0.11*	0.04	0.05	0.18**	0.16**	−0.09*	−0.11*	0.14**	0.13**	0.15**	0.15**	0.65**	(0.76)										
(15) Workload T1	2.31	0.59	−0.34**	−0.25**	−0.14**	−0.15**	–0.00	–0.02	−0.25**	−0.18**	0.18**	0.14**	0.19**	0.11*	0.35**	0.25**	(0.87)									
(16) Workload T2	2.24	0.56	−0.22**	−0.32**	−0.13**	−0.12**	0.03	0.02	−0.18**	−0.22**	0.19**	0.23**	0.12**	0.17**	0.19**	0.28**	0.64**	(0.87)								
(17) Autonomy T1	2.81	0.61	0.25**	0.22**	0.05	–0.02	−0.06*	–0.04	0.13**	0.11**	−0.37**	−0.41**	–0.04	–0.05	–0.05	−0.12**	−0.08**	–0.08	(0.86)							
(18) Autonomy T2	2.78	0.62	0.19**	0.25**	0.02	–0.00	–0.01	–0.02	0.09*	0.17**	−0.38**	−0.38**	–0.07	–0.05	−0.10*	−0.12**	–0.02	–0.07	0.71**	(0.89)						
(19) Supervisor support T1	3.16	0.78	0.12**	0.09**	0.12**	0.05	0.01	0.03	0.10**	0.06*	−0.08**	0.00	0.025	–0.06	–0.01	0.00	−0.11**	0.00	0.17**	–0.00	(0.87)					
(20) Supervisor support T2	3.36	0.57	0.26**	0.32**	0.13**	0.20**	0.00	0.03	0.24**	0.33**	−0.28**	−0.26**	–0.01	0.03	−0.20**	−0.19**	−0.23**	−0.23**	0.28**	0.28**	0.08	(0.81)				
(21) Colleague support T1	3.21	0.77	0.08**	0.07*	0.07**	–0.00	0.01	0.03	0.06*	0.03	–0.05	0.05	0.06*	–0.05	0.04	0.02	–0.05	0.03.3	0.09**	–0.03	0.76**	–0.01	(0.80)			
(22) Colleague support T2	3.41	0.48	0.22**	0.21**	0.02	0.03	0.03	–0.03	0.14**	0.21**	0.03	0.04	0.03	0.10*	−0.12**	−0.11**	−0.20**	−0.16**	0.10*	0.14**	–0.01	0.32**	0.07	(0.79)		
(23) Future time perspective	3.03	0.85	0.29**	0.28**	0.43**	0.38**	0.45**	0.46**	0.14**	0.13**	−0.12**	−0.17**	0.04	0.01	0.04	0.08	–0.04	–0.02	0.11**	0.11**	0.11**	0.12**	0.07*	0.06	(0.84)	
(24) Age	46.79	11.06	−0.18**	−0.19**	−0.28**	−0.27**	−0.35**	−0.41**	0.09**	0.08**	0.06*	0.03	–0.01	0.02	−0.08**	−0.11*	0.05	0.04	0.02	0.04	–0.05	–0.01	-/04	–0.02	−0.65**	

**p < 0.01, *p < 0.05.

As shown in Table 3, work ability at the first measurement moment was positively associated with work ability measured at the second measurement moment (β = 0.733, *p* < 0.01; *R*^2^ = 0.537). In the second step, future time perspective, age, the job demands, and job resources at T1 were added to the model. After controlling for the other variables in the model, future time perspective remained positively associated with work ability (T2; β = 0.103, *p* < 0.01), whereas age was not associated with work ability (β = −0.020, *p* = 0.49). None of the job demands and job resources were significantly related to work ability. Adding the job demands and job resources at T2 to this model led to a decrease in explained variation of 17.4%. Therefore, we decided to not add job demands and job resources at T2 to this model. The second model explained 3.0% additional variation. In the third model, the interaction terms of job demands with age, job demands with future time perspective, job resources with age, and job resources with future time perspective were added. None of these interactions were significant. Overall, model 3 resulted in the highest explained variance. However, chi square difference tests suggest that model 2 (the model with control variables, age, and future time perspective, job demands and job resources) reveals the best fit to our data, and that adding the interactions between age and future time perspective and job demands and job resources have no significant added value in explained variance. These results indicate that future time perspective, and not age, job demands, or job resources, is particularly important for stimulating work ability of the healthcare workers over time.

**TABLE 3:** Standardized results with work ability at T2 as an outcome variable.

**Model 1**	**Model 2**	**Model 3**	**β**	**p**	**β**	**p**	**β**	**p**
**Model 1: control variable**								
Work ability T1			0.73	0.00	0.69	0.00	0.68	0.00
**Model 2: control variable, age, future time perspective, job demands, and job resources**								
Future time perspective					0.10	0.00	0.30	0.37
Age					–0.03	0.39	0.03	0.94
Physical demands					–0.05	0.07	0.23	0.29
Mental demands					–0.02	0.30	–0.03	0.87
Emotional demands					–0.03	0.20	–0.21	0.37
Workload					–0.00	0.89	–0.07	0.77
Autonomy					0.02	0.45	0.03	0.89
Supervisor support					0.02	0.57	0.48	0.18
Colleague support					–0.03	0.37	–0.12	0.74
**Model 3: control variable, age, future time perspective, job demands, job resources, and interactions**								
Future time perspective * physical demands							–0.09	0.48
Future time perspective * mental demands							–0.13	0.56
Future time perspective * emotional demands							0.08	0.69
Future time perspective * workload							0.08	0.64
Future time perspective * autonomy							–0.05	0.82
Future time perspective * supervisor support							–0.18	0.51
Future time perspective * colleague support							–0.00	0.99
Age * physical demands							–0.24	0.13
Age * mental demands							0.14	0.56
Age * emotional demands							0.21	0.34
Age * workload							0.02	0.92
Age * autonomy							0.01	0.95
Age * supervisor support							–0.47	0.12
Age * colleague support							0.12	0.68
R-square			0.537		0.567		0.572	
R-square increase					0.030		0.005	
Chi-square difference test:					63.039(9)	0.00	−10.77(14)	0.70

As shown in Table 4, vitality at the first measurement moment is positively associated with vitality measured at the second measurement moment (β = 0.675, *p* < 0.01; *R*^2^ = 0.431). In the second step, future time perspective, age, job demands, and job resources at T1 and T2 were added as predictors of vitality at the second measurement moment. After controlling for the other variables in the model, future time perspective (β = 0.188, *p* < 0.01) is positively associated with vitality. Of all job demands and job resources only colleague support was significantly related to vitality at T1 (β = 0.172, *p* = 0.01) and T2 (β = 0.166, *p* < 0.01), when accounting for the other variables in the model. This second model explained 20.7% additional variation. In the third model, the interactions terms were added. None of the interactions were significant. Adding these interactions led to a decrease of 23.9% in explained variation. Overall, model 2 (the model with control variables, age, future time perspective, job demands, and job resources at T1 and T2) has the highest explained variance. Moreover, chi square difference tests suggest that model 2 has the best fit and that adding the interactions with age and future time perspective and job demands and job resources has no significant added value in explained variance. These results indicate that future time perspective and collegial support are particularly important for stimulating vitality over time.

**TABLE 4:** Standardized results with vitality at T2 as an outcome variable.

**Model 1**	**Model 2**	**Model 3**	**β**	**p**	**β**	**p**	**β**	**p**
**Model 1: control variable**								
Vitality T1			0.66	0.00	0.71	0.00	0.71	0.00
**Model 2: control variable, age, future time perspective, job demands, and job resources**								
Future time perspective					0.19	0.00	1.10	0.02
Age					0.03	0.51	–0.37	0.46
Physical demands T1					–0.16	0.23	–0.17	0.40
Mental demands T1					0.24	0.18	0.15	0.47
Emotional demands T1					–0.06	0.47	0.36	0.10
Workload T1					–0.03	0.81	0.18	0.50
Autonomy T1					0.01	0.96	–0.09	0.70
Supervisor support T1					–0.11	0.22	0.01	0.95
Colleague support T1					0.34	0.01	0.05	0.81
Physical demands T2					0.03	0.81	0.01	0.85
Mental demands T2					–0.01	0.94	–0.02	0.72
Emotional demands T2					–0.04	0.60	–0.01	0.80
Workload T2					–0.02	0.91	–0.09	0.44
Autonomy T2					0.00	0.97	0.03	0.72
Supervisor support T2					–0.05	0.60	–0.04	0.57
Colleague support T2					0.17	0.01	0.17	0.01
**Model 3: control variable, age, future time perspective, job demands, job resources, and interactions**								
Future time perspective * physical demands							–0.11	0.73
Future time perspective * mental demands							–0.54	0.12
Future time perspective * emotional demands							–0.27	0.33
Future time perspective * workload							–0.10	0.69
Future time perspective * autonomy							–0.15	0.56
Future time perspective * supervisor support							–0.10	0.55
Future time perspective * colleague support							0.19	0.35
Age * physical demands							0.34	0.38
Age * mental demands							0.41	0.21
Age * emotional demands							–0.52	0.08
Age * workload							–0.11	0.53
Age * autonomy							0.22	0.41
Age * supervisor support							–0.05	0.83
Age * colleague support							0.06	0.81
R-square			0.431		0.603		0.364	
R-square increase					0.172		–0.239	
Chi-square difference test:					665.12(16)	0.00	−15.81(14)	0.37

As shown in Table 5, internal employability at the first measurement moment was positively associated with internal employability measured at the second measurement moment (β = 0.675, *p* < 0.01; *R*^2^ = 0.373). In the second model, future time perspective, age, job demands, and job resources at T1 and T2 were added as predictors of internal employability at the second measurement moment. After accounting for the other variables in the model, future time perspective and age were not related to internal employability, whereas autonomy at T1 (β = 0.189, *p* = 0.04) was positively related to internal employability, and workload at T2 (β = −0.304, *p* = 0.05) and autonomy at T2 (β = −0.247, *p* = 0.00) were significantly negatively related to internal employability. This second model explained 21.8% additional variation. In the third model, the interactions terms were added. None of the interactions were significant. Adding these interactions led to a 4.3% increase in explained variation. Overall, model three has the highest explained variance. However, chi-square difference tests revealed that the second model (with internal employability at baseline, age, future time perspective, job demands, and job resources at T1 and T2 as predictors) fits the data best, indicating that work characteristics are important factors for internal employability over time.

**TABLE 5:** Standardized results with internal employability at T2 as an outcome variable.

**Model 1**	**Model 2**	**Model 3**	**β**	**p**	**β**	**p**	**β**	**p**
**Model 1: control variable**								
Internal employability T1			0.66	0.00	0.83	0.00	0.53	0.00
**Model 2: control variable, age, future time perspective, job demands, and job resources**								
Future time perspective					0.05	0.33	0.15	0.82
Age					–0.05	0.32	0.11	0.86
Physical demands T1					–0.16	0.35	0.12	0.64
Mental demands T1					–0.09	0.67	–0.23	0.34
Emotional demands T1					0.09	0.39	0.06	0.80
Workload T1					0.19	0.28	0.45	0.16
Autonomy T1					0.29	0.04	0.25	0.37
Supervisor support T1					–0.07	0.55	0.01	0.98
Colleague support T1					0.27	0.07	–0.23	0.39
Physical demands T2					0.16	0.31	0.07	0.44
Mental demands T2					–0.01	0.97	0.02	0.81
Emotional demands T2					–0.07	0.48	–0.04	0.46
Workload T2					–0.35	0.05	–0.26	0.09
Autonomy T2					–0.39	0.00	–0.23	0.01
Supervisor support T2					–0.07	0.55	–0.02	0.79
Colleague support T2					0.27	0.07	0.12	0.10
**Model 3: control variable, age, future time perspective, job demands, job resources, and interactions**								
Future time perspective * physical demands							0.38	0.38
Future time perspective * mental demands							–0.14	0.74
Future time perspective * emotional demands							–0.62	0.08
Future time perspective * workload							–0.53	0.09
Future time perspective * autonomy							0.15	0.60
Future time perspective * supervisor support							0.37	0.14
Future time perspective * colleague support							0.10	0.70
Age * physical demands							–0.79	0.08
Age * mental demands							0.42	0.28
Age * emotional demands							0.66	0.08
Age * workload							0.08	0.69
Age * autonomy							–0.24	0.44
Age * supervisor support							–0.31	0.23
Age * colleague support							0.08	0.78
R-square			0.373		0.591		0.634	
R-square increase					0.218		0.043	
Chi-square difference test:					521.81(16)	0.00	−14.45(14)	0.42

**FIGURE 1:**
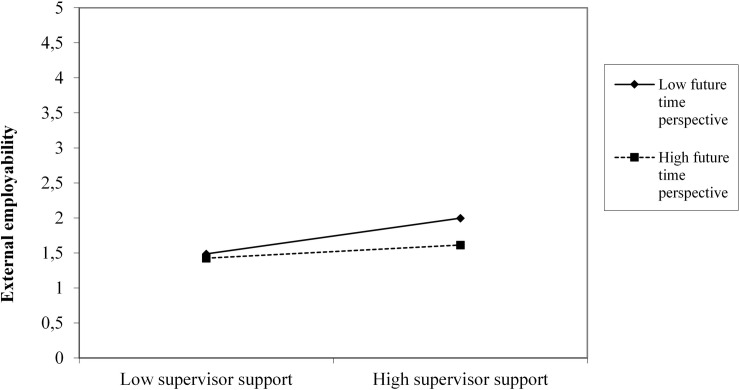
The interaction effect between supervisor support and future time perspective on external employability.

As shown in Table 6, external employability at the first measurement moment was positively associated with external employability measured at the second measurement moment (β = 0.750, *p* < 0.01; *R*^2^ = 0.562). In the second model, future time perspective, age, job demands, and job resources at T1 were added as predictors of external employability at the second measurement moment. After controlling for the other variables in the model, future time perspective was positively associated with external employability (β = 0.086, *p* = 0.00), whereas age was negatively related to external employability (β = −0.113, *p* = 0.00). Of the job demands and job resources only emotional demands was significantly positively related to external employability (β = 0.050, *p* = 0.01) after adjusting for the other variables. However, adding job demands and job resources at T2 led to a decrease in explained variation of 10.1%. We therefore decided not to add job demands and job resources at T2 to the model. The final second model explained 2.8% more variation than the previous model. In the third model, the interactions terms were added. Only the interaction between future time perspective and supervisor support was significant (β = −0.462, *p* = 0.03). The interaction (see Figure 1) revealed that high levels of supervisor support and closed future time perspective was related to higher external employability (partial support hypothesis 6). Adding these interactions led to a 0.5% increase in explained variation. Overall, model 3 resulted in the highest explained variance. However, the chi square test revealed that model 1 fitted the data best, indicating future time perspective and supervisory support is important for external employability over time.

**TABLE 6:** Standardized results with external employability at T2 as an outcome variable.

**Model 1**	**Model 2**	**Model 3**	**β**	**p**	**β**	**p**	**β**	**p**
**Model 1: control variable**								
External employability T1			0.75	0.00	0.66	0.00	0.66	0.00
**Model 2: control variable, age, future time perspective, job demands, and job resources**								
Future time perspective					0.09	0.00	0.21	0.39
Age					–0.11	0.00	0.03	0.89
Physical demands					0.04	0.05	–0.06	0.74
Mental demands					–0.02	0.25	–0.05	0.78
Emotional demands					0.05	0.01	–0.14	0.45
Workload					–0.03	0.20	0.22	0.25
Autonomy					0.00	0.97	0.24	0.19
Supervisor support					–0.00	0.95	0.46	0.08
Colleague support					0.02	0.59	–0.33	0.23
**Model 3: control variable, age, future time perspective, job demands, job resources, and interactions**								
Future time perspective * physical demands							–0.04	0.71
Future time perspective * mental demands							0.07	0.70
Future time perspective * emotional demands							0.22	0.16
Future time perspective * workload							–0.19	0.17
Future time perspective * autonomy							–0.15	0.32
Future time perspective * supervisor support							–0.46	0.03
Future time perspective * colleague support							0.32	0.13
Age * physical demands							0.15	0.22
Age * mental demands							–0.01	0.97
Age * emotional demands							0.11	0.53
Age * workload							–0.17	0.29
Age * autonomy							–0.23	0.17
Age * supervisor support							–0.46	0.28
Age * colleague support							0.19	0.40
R-square			0.562		0.590		0.595	
R-square increase					0.028		0.005	
Chi-square difference test:					12.62(9)	0.18	−15.88(14)	0.32

## Discussion

The current two-wave complete panel study was the first longitudinal study to examine the dynamics between age, future time perspective, specific job demands and job resources and indicators of sustainable employability in a healthcare context. Based on earlier lifespan developmental and psychosocial work theories, we formulated and tested different hypotheses in a unique complete panel of 1478 healthcare workers. We found mixed results for our hypotheses. More specifically, only a negative relation was found between calendar age and external employability, and no significant relations were found between calendar age and other indicators of sustainable employability (limited support hypothesis 1).

More consistent positive significant relations were found between an open future time perspective and across-time changes in work ability, vitality as well as external employability (supporting hypothesis 2). In contrast to our expectations, the current results did not explain more variance in sustainable employability by including and testing for specific types of job demands (like physical versus emotional demands). As only significant negative relations were found between workload and internal employability (limited support hypothesis 3), and positive relations were found between emotional demands and internal employability (in contrast with hypothesis 3). Finally, we found more consistent significant positive relations between colleague support and vitality, and a significant positive relation between job autonomy and internal employability (partial support for hypothesis 4).

Furthermore, our explorative test of possible moderating effects of age and future time perspective in predicting relations between psychosocial work characteristics and indicators of sustainable employability revealed only one significant interaction effect in line with Hypothesis 6 (rejecting Hypothesis 5). We found a significant interaction between supervisor support and future time perspective in explaining across-time changes in external employability (partial support hypothesis 6). This shows that a supportive work climate, and an open future time perspective can play an important role in sustaining the reported external employability levels of aging healthcare workers.

The negative relation between calendar age and across-time changes in external employability is consistent with earlier research in different sectors indicating the risk of labor market age stereotyping. For example, Van Vuuren et al. (2011) found in their cross-sectional survey study among teachers similar significant negative relations between calendar age and labor-market based external employability. In their longitudinal survey study among 284 low-qualified employees of 35 different companies, Raemdonck et al. (2008) also found that higher calendar age was related to reduced job mobility, vertical mobility, as well as reduced job turnover. It is important to further monitor and examine the negative relation between calendar age and external employability of healthcare workers to make sure aging workers do not suffer from less chances of vertical or horizontal external mobility to facilitate their sustainable employability across time.

Fortunately, we also found consistent positive and buffering effects of future time perspective in relation to the external employability of healthcare workers. Consequently, the current study demonstrates the importance of broadening the future time perspective in predicting across-time changes in the sustainable employability of aging healthcare workers, indicating the importance of taking a life-span perspective in relations between aging and work ability (Pak et al., 2018; Rudolph et al., 2018; Zacher et al., 2018a). It is important to further investigate the relations between future time perspective, supervisor support and indicators of sustainable employability using different samples and investigating different professions in healthcare contexts. Future research can further examine the effects of time-broadening interventions in sustaining or positively influencing the external employability of healthcare workers.

As we found a meaningful interaction between future time perspective and high supervisor support in maintaining higher levels of external employability of healthcare workers, new studies can also examine the dynamics between supervisor support (in terms of communication and behavior) in broadening temporal horizons or perspectives of healthcare workers. For example, recent research of Nielsen and Taris (2019) points to the accumulating evidence for an association between leadership or supervisor behavior and positive mental well-being, but no study to date has examined the effects of leaders in developing time-broadening perspectives or workplans for aging healthcare workers (see also Thun and Bakker, 2018; Nikolova et al., 2019). Support from leaders and colleagues can have a positive effect on workers’ well-being (Nielsen and Taris, 2019). The current study suggests that high levels of supervisor support can form a significant buffer for maintaining higher levels of external employability in the case of a low future time perspective at work. Unfortunately, older workers are often offered less opportunities for training and development by their supervisors (Furunes and Mykletun, 2010; Truxillo et al., 2012, 2015). Some managers lower the demands on older workers as a way of sustaining their work ability, whereas this action from a worker perspective can also be seen as age discriminatory practices (Furunes and Mykletun, 2010; Truxillo et al., 2015), and thus limits their future time perspective (Rudolph et al., 2015, 2018). From a scientific as well as practical perspective it is therefore important to further examine the role of supervisors can play in broadening the time perspective as well as sustainable employability of aging healthcare workers.

### Limitations

The current study investigated all healthcare workers as one group and did not differentiate in job functions because the institutions involved used different job function indicators and job titles. The current design only allowed for a gross categorization, dividing healthcare workers from staff personnel. Thus, we conducted *post hoc* tests to test for possible differences between support staff versus healthcare workers in the variables under study. The results indicated no significant differences in results found per hypothesis or for the outcomes under study. Nonetheless, future research may further examine potential influences of subgroup specific characteristics on healthcare workers’ sustainable employability.

Second, the measurement points in this study are 6 months apart. Dormann and Griffin (2015) recommend using shorter time lags in panel studies, and we therefore think the chosen time-lag was appropriate for the concepts included in our research. Nonetheless, as relatively few effects were found for the included job demands and job resources to explain across-time changes in sustainable employability, longer lengths of time-lags and 3 or more time points across time may reveal additional effects for our formulated hypotheses and variables under study (see also De Lange et al., 2004). Third, our work has been based on subjective survey measures, resulting in the risk of common-method bias (Steiger and Lind, 1980). Testing multiple competing models in a longitudinal complete panel design and controlling for autocorrelations aimed to lessen these risk of common-method bias (De Lange et al., 2009). Nonetheless, future studies can study changes in a long-term perspective using mixed method designs to further examine the causal nature of relations between age-related variables, psychosocial work characteristics and work outcomes of healthcare workers.

Finally. though the WAI measures of work ability are conceptualized as a multidimensional construct, we treated work ability as a unidimensional construct in the study in accordance with the way it is applied and interpreted in the health care sector in many countries such as Finland and Netherlands (see Osagie et al., 2019 for a review). However, it would be interesting and relevant to explore the influences of the predictors on specific indicators of work ability in future studies among healthcare workers.

### Originality/Value

The current longitudinal complete panel study was the first multi-wave study to examine relations between aging, time perspective and indicators of sustainable employability in healthcare work. Accordingly, the results of our two-wave complete panel study provide new insights into the question how to sustain aging workers in healthcare. This is imperative as an aging global workforce can present healthcare organizations with untapped opportunities. Healthcare organizations that plan, design and find management approaches to prolong working lives of older workers can reduce potential liability concerns and costs of reduced performance or disability pensions (Bakker, 2018; Herkes et al., 2019). Creating ways for healthcare workers to have meaningful, productive multi-stage and multidimensional careers is a major opportunity to proactively engage workers within healthcare (Stuer et al., 2019).

As talent markets grow more competitive, and employers in the healthcare sector have more and more difficulties in recruiting and retaining enough competent staff, healthcare organizations can find it valuable to keep aging workers in their jobs across time and facilitate their sustainable employability (Truxillo et al., 2015). Based on the results of the current longitudinal study, we can conclude that by broadening and developing meaningful time horizons at work and creating supportive work environments for aging workers, we may be better able to retain healthcare workers at work to ensure a sufficient level of quality of healthcare (Teoh et al., 2019).

## Data Availability Statement

All datasets generated for this study are included in the article/supplementary material.

## Ethics Statement

The studies involving human participants were reviewed and approved by Ethical Commision University of Tilburg, Tilburg, Netherlands. The patients/participants provided their written informed consent to participate in this study.

## Author Contributions

All authors listed have made a substantial, direct and intellectual contribution to the work, and approved it for publication.

## Conflict of Interest

The authors declare that the research was conducted in the absence of any commercial or financial relationships that could be construed as a potential conflict of interest.
